# Mineralocorticoid receptor antagonists for chronic heart failure: a meta-analysis focusing on the number needed to treat

**DOI:** 10.3389/fcvm.2023.1236008

**Published:** 2023-11-06

**Authors:** Chang Geng, Yu-Cheng Mao, Su-fen Qi, Kai Song, Hong-Fei Wang, Zi-yan Zhang, Qing-Bao Tian

**Affiliations:** Hebei Key Laboratory of Environment and Human Health, Department of Epidemiology and Statistics, School of Public Health, Hebei Medical University, Shijiazhuang, China

**Keywords:** mineralocorticoid receptor antagonists, chronic heart failure (CHF), cardiovascular—history, number needed to treat (NNT), meta-analysis

## Abstract

**Aims:**

Recent studies have shown that mineralocorticoid receptor antagonists (MRAs) can decrease mortality in patients with heart failure; however, the application of MRAs in current clinical practice is limited because of adverse effects such as hyperkalemia that occur with treatment. Therefore, this meta-analysis used the number needed to treat (NNT) to assess the efficacy and safety of MRAs in patients with chronic heart failure.

**Methods:**

We meta-analysed randomized controlled trials (RCTs) which contrasted the impacts of MRAs with placebo. As of March 2023, all articles are published in English. The primary outcome was major adverse cardiovascular events (MACE), and secondary outcomes included all-cause mortality, cardiovascular death, myocardial infarction (MI), stroke, and adverse events.

**Results:**

We incorporated seven studies with a total of 9,056 patients, 4,512 of whom received MRAs and 4,544 of whom received a placebo, with a mean follow-up period of 2.1 years. MACE, all-cause mortality, and cardiovascular mortality were all reduced by MRAs, with corresponding numbers needed to treat for benefit (NNTB) of 37, 28, and 34; as well as no impact on MI or stroke. MRAs increased the incidence of hyperkalemia and gynecomastia, with the corresponding mean number needed to treat for harm (NNTH) of 18 and 52.

**Conclusions:**

This study showed that enabling one patient with HF to avoid MACE required treating 37 patients with MRAs for 2.1 years. MRAs reduce MACE, all-cause mortality, and cardiovascular death; however, they increase the risk of hyperkalemia and gynecomastia.

## 1. Introduction

One of the most prevalent public health issues of the 21st century is chronic heart failure (CHF) (1). Any structural or functional change, such as ventricular filling or blood ejection, is responsible for the onset and progression of heart failure (HF), a complex, clinically integrated disease (2). The prevalence of heart failure (HF) is increasing every year due to an ageing population and ongoing advancements in medical technology (3). Although much progress has been made with the management of patients suffering from HF, their prognosis remains unfavorable with high rates of hospitalization and mortality due to the devastating nature of HF (4). To further reduce mortality and morbidity and enhance HF patients' quality of life (QOL), new treatment strategies must be developed.

The role of Mineralocorticoid receptor (MR) is to maintain the Na+/K + balance in the nephron (5). It is mostly expressed in the heart (cardiomyocytes, fibroblasts, blood vessels) (6). Over-activation adversely affects the heart and kidneys, promoting inflammation and fibrosis as part of the pathogenesis of several cardiovascular and renal illnesses (7). In contrast, mineralocorticoid receptor antagonists (MRAs) can inhibit this receptor, thereby reducing the development of related diseases (8). MRAs can be classified as either steroidal MRAs (e.g., spironolactone, canrenone, eplerenone) or non-steroidal MRAs (e.g., finerenone). Canrenone is thought to be the main active metabolite of spironolactone (9). Eplerenone, on the other hand, is more selective, reducing non-targeted binding to progesterone or androgen receptors and therefore reducing the incidence of sexual adverse reactions, but is less binding to MR. A recently studied non-steroidal MRA, finerenone, however, binds MR in a different way to other steroidal MRAs, activates different gene pathways, and may attenuate aldosterone-induced hemodynamic and pro-fibrotic damage, thereby reducing some of the side effects seen in steroidal MRA treatment (10). MRAs have a proven performance history in reducing heart failure and have become one of the recommended modalities for the treatment of HF (11). Although the results of randomized controlled trials provide relatively reliable results, combining trials and meta-analyzing them may well provide stronger statistical power to demonstrate efficacy.

The Number Needed to Treat (NNT) represents a clinical efficacy standard that quantifies the effectiveness of pharmacological interventions within specific ranges, providing clarity of interpretation for physicians and patients (12). In addition, healthcare organizations can utilize this metric as a benefit-risk assessment tool to develop drug use strategies (13, 14). NNT values, in contrast to relative risk, are unaffected by sample size and, as a result, have no effect on *p*-values, which are used to reflect and explain clinical implications. Lower NNT values indicate greater effectiveness of the medication (15, 16).

Therefore, in this meta-analysis, we applied NNT to examine the risks and benefits associated with the treatment of people with chronic heart failure by using mineralocorticoid receptor antagonists.

## 2. Methods

### 2.1. Trial selection

We conducted a search of PubMed, Web of Science, the Cochrane Library, and ClinicalTrials.gov website, starting with the database until March 2023. MeSH terms were used for the search, and related terms include: heart failure, mineralocorticoid receptor antagonists, spironolactone, canrenone, eplerenone, finerenone, randomized controlled trials.

The following criteria must be met for inclusion: (1) randomized controlled trials (RCTs) (2) comparison with placebo (3) participants with chronic heart failure (4) use of MRAs (spironolactone, canrenone, eplerenone or finerenone) (5) adult patients (age 》18) (6) follow-up > 1 year (7) at least one outcome reported (8) sample size >100 cases.

Of the 2,601 articles identified, 7 met our inclusion criteria.

### 2.2. Data extraction and quality assessment

Two authors separately analyzed the risk of bias for the incorporated trials and extracted the required data for generalisation. Extract relevant information including subjects (number of participants, mean follow-up time, mean age at baseline), intervention (type of MRAs), control (placebo), and efficacy outcomes. The risk of bias was evaluated by applying a tool produced by the Cochrane Collaboration. Discrepancies, if any, were resolved by discussion.

### 2.3. Endpoints definition

We compared the outcomes of patients treated with MRAs with those not treated with MRAs. The primary outcome was major adverse cardiovascular events (MACE), including cardiovascular death, myocardial infarction (MI) and stroke. Secondary endpoints were all-cause mortality, cardiovascular death, MI, stroke, and adverse events. Adverse events include mainly hyperkalemia and gynecomastia.

### 2.4. Statistical analysis

It is more robust to use relative indices to calculate NNTs; therefore, we obtained pooled efficacy and NNTs by calculating risk ratios (RRs). The Mantel-Haenszel random effects model to measure and aggregate the RR was utilized, considering the proportion of each trial to the total. The *I*^2^ statistic was used to measure heterogeneity; an *I*^2^ of less than 25% was deemed minimal, a value between 25% and 50% was deemed moderate, and an *I*^2^ of greater than 50% was deemed large. The following formula was utilized when calculating NNT: NNT = 1/[(1-RR)CER], where CER is the range of 0 to 1 for the control (placebo) event rate (12). Supplementary Table S1 contains the CERs used to calculate the NNT values for each summary (17). To rationalize the interpretation of NNTs, NNTs are taken as integers. Assuming the 95% CI for NNTs crossed positive endlessness, this intended that there was no statistical significance. Due to differences in follow-up time, NNTs may be biased when compared between four distinct drug classes (spironolactone, canrenone, eplerenone, and finerenone) at a given outcome. Therefore, we normalized the follow-up time using the formula suggested by Sackett et al. (18). The formula is as follows. NNT:T × T ÷ S = NNT:S, (NNT:S is the rectified NNT, NNT:T is the real calculated to NNT, T is the real duration of follow-up and S is the normalized number of years). NNT typically decreases with increasing time since treatment began. In this equation, both the outcome incidence and the treatment effect of the drug are set to remain constant, despite changes in time.

In addition, we hypothesized that MACE might be associated with mean age, year of publication, mean follow-up time, percentage of male participants, and body mass index (BMI). To assess this hypothesis, a random effect univariate meta-regression was conducted. If the 2-tailed *p*-value was <0.05, it was regarded as statistically significant.

All analyses for the meta-analysis were calculated using Review Manager V.5.4.1 (RevMan), R software V.4.2.1, and Stata, V.16.0.

## 3. Results

### 3.1. Study characteristics

**Figure 1:**
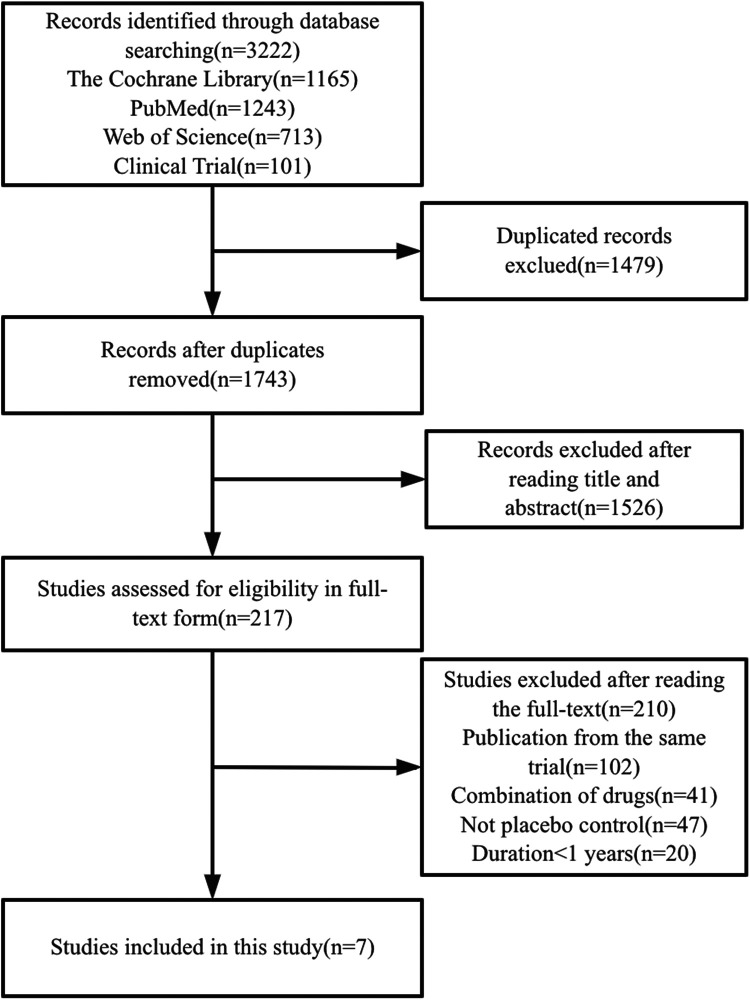
Flowchart of study selection for meta-analysis.

At the first time there were 3,222 records read. Among them, 3,215 were excluded because there were no associated results, the identical trial was published, lasted <1 year, had <100 patients, was not a randomized controlled trial, was not placebo-controlled, or was repeatedly published. Ultimately, there were seven trials enrolled in this meta-analysis (Figure 1) with a population of 9,056 patients and a mean follow-up of 2.1 years (range 1–3.7 years) for the included trials (19–25). Four trials (*n* = 5,660) compared spironolactone with placebo, one trial (*n* = 438) compared canrenone with placebo treatment, and two trials (*n* = 2,958) compared eplerenone with placebo treatment. Their overall characteristics are shown in Table 1. The majority from the study were men with a mean age of 68 years. The total quality of these studies was great. (Supplementary Figure S1).

**Table 1:** Characteristics of interventions and populations at baseline included RCT.

Author	Year	No. of patients	Drugs		Baseline characteristics						
			Treatment	Control	Mean age, year	Men, %	Follow-up, year	SBP (mmHg)	DBP (mmHg)	BMI (kg/m^2^)	Heart rate (bpm)
Vizzardi	2014	130	Spironolactone	Placebo	62.0	NA	3.70	121.0	67.0	25.75	67.3
Tsutsui	2018	221	Eplerenone	Placebo	68.7	79.6	2.00	117.6	69.5	22.6	74.3
Preiss	2012	2,737	Eplerenone	Placebo	68.6	77.7	1.75	124.1	74.6	27.5	71.7
Boccanelli	2008	438	Canrenone	Placebo	62.5	83.5	1.00	128.0	NA	26.8	66.8
Edelmann	2013	422	Spironolactone	Placebo	67.0	48.0	1.00	135.0	79.0	28.9	65.0
Pitt	1999	1,663	Spironolactone	Placebo	65.0	73.0	2.00	122.0	75.0	NA	81.0
Pitt	2014	3,445	Spironolactone	Placebo	68.7	48.5	3.30	130.0	80.0	31.0	68.0

SBP, systolic blood pressure; DBP, diastolic blood pressure; BMI, body mass index.

### 3.2. End points

#### 3.2.1. Major adverse cardiovascular events

**Figure 2:**
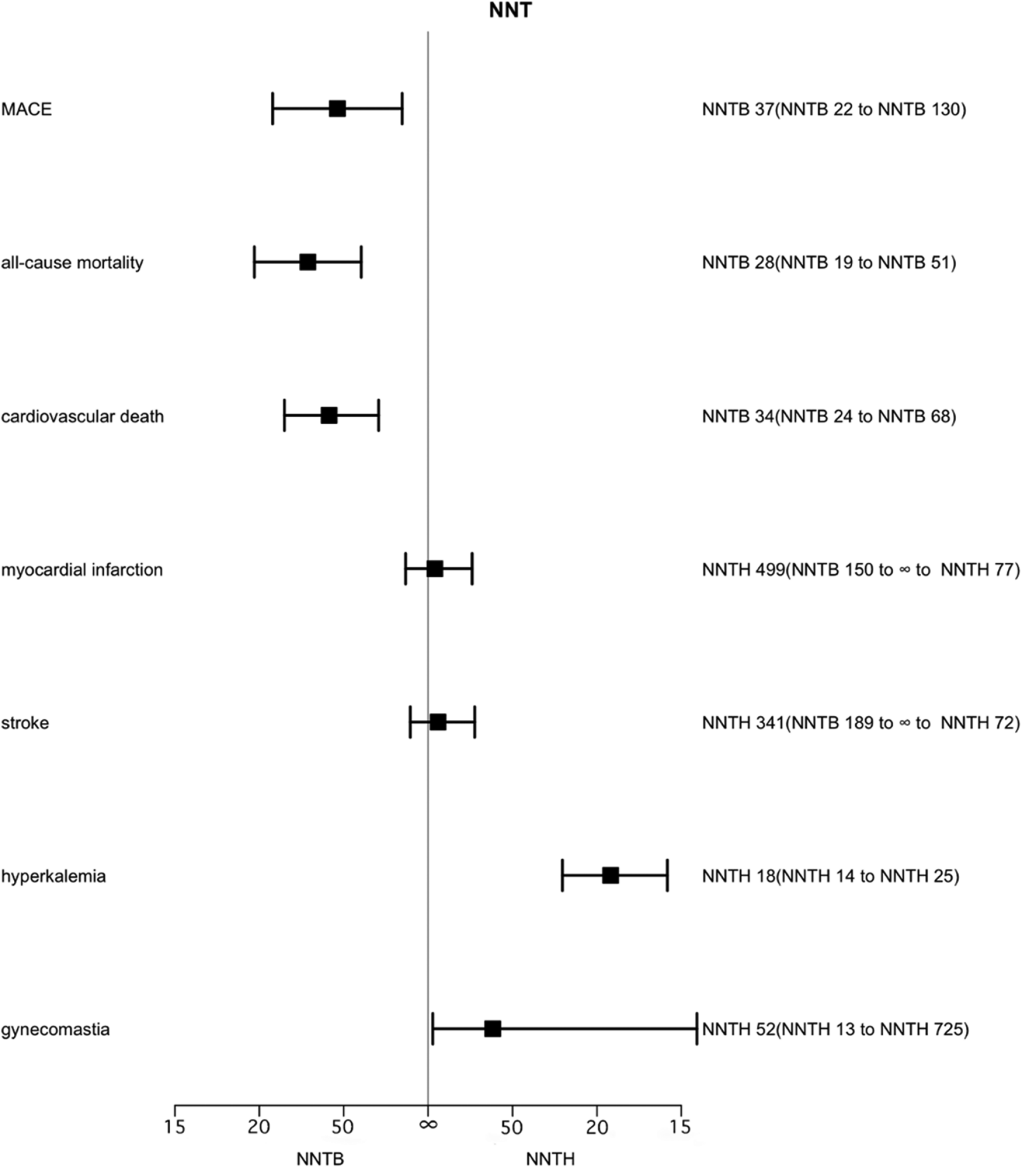
Effect of MRAs on major adverse cardiovascular events, all-cause mortality, cardiovascular death, myocardial infarction, stroke, hyperkalemia and gynecomastia over 2.1 years. Number needed to treat (NNT) with the corresponding confidence intervals (CIs). MACE, major adverse cardiovascular events; NNT, the number needed to treat; NNTB, number needed to treat to benefit; NNTH, number needed to treat to harm.

In total, 7 studies evaluated the effect of MRAs on MACE in a total of 9,056 patients. Patients who were allocated to either the MRAs or the placebo group, NNT for MACE was 37 (95% CI, NNTB 22 to NNTB 130), that is, in order to avoid a single MACE, 37 patients needed to receive MRAs for 2.1 years (Figure 2). Low heterogeneity existed between trials (*I*^2 ^= 22%) (Supplementary Figure S2).

**Figure 3:**
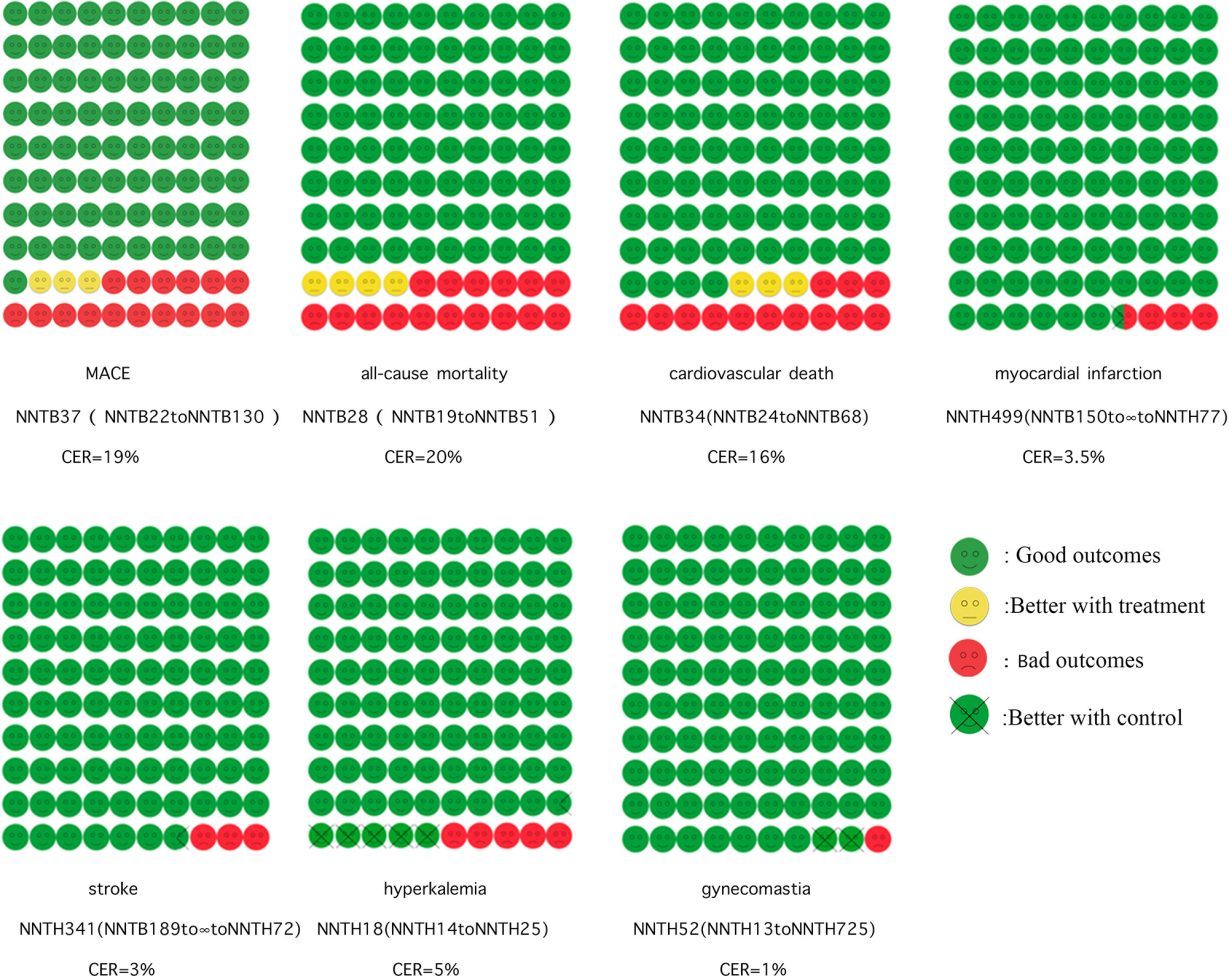
Cates plot. Shows the effect of MRAs on major adverse cardiovascular events, all-cause mortality, cardiovascular death, myocardial infarction, stroke, and hyperkalemia. The 100 smiley faces represent 1,000 participants treated with MRAs. Yellow faces indicate that no outcome event occurred if treated with MRAs. Green faces mean no outcome event occurred even without treatment with MRAs. Red faces mean that the outcome event will occur even if treated with MRAs. Crossed green faces indicate that the patient did not reach the outcome event with the control group.

The role of MRA in MACE is shown in Figure 3. For a more visual comparison, we created cates plots by converting the NNT from the number of people treated needed to protect an outcome event to the number who were protected from an outcome event at 2.1 years of treating 1,000 people. Altogether 7 studies were enrolled, with a CER of 19.2% and an average of 27–28 beneficiaries out of 1,000 patients treated with MRAs.

#### 3.2.2. All-cause mortality

Seven studies assessed the effect of MRAs on all-cause mortality in 9,056 patients. MRAs significantly reduced all-cause mortality (RR 0.82, 95% CI 0.74–0.90), with low heterogeneity (*I*^2 ^= 9%) (Supplementary Figure S3). Among patients randomized to the MRAs and placebo groups, 28 patients had to take MRAs for 2.1 years to avoid one all-cause mortality (95% CI, NNTB 19 to NNTB 51). When translated into the number of adverse events preventable by treating 1,000 people, treatment with MRA resulted in 36–37 avoided deaths.

#### 3.2.3. Cardiovascular death

Six trials involving 8,634 patients reported the effect of MRAs on cardiovascular mortality in patients. The effect of MRAs on cardiovascular death was statistically significant (RR 0.80, 95% CI 0.71–0.90), with low heterogeneity (*I*^2 ^= 15%) (Supplementary Figure S4). In patients randomized to the MRAs and placebo groups, the NNT for cardiovascular death was 34 (95% CI, NNTB 24 to NNTB 68) and 34 patients had to be used with MRAs for 2.1 years to have one cardiovascular death averted. Alternatively, treating 1,000 patients with MRA for 2.1 years could benefit 29–30 people.

#### 3.2.4. Myocardial infarction

The analysis of MI included 4 studies (5,751 patients). The corresponding NNTH for MRAs was 499 (95% CI, NNTB 150 to ∞ to NNTH 77). No significant differences were found in MI between MRAs and controls (RR 1.06, 95% CI 0.80–1.39), with no heterogeneity (*I*^2 ^= 0%, *p* = 0.69) (Supplementary Figure S5).

#### 3.2.5. Stroke

Four examinations (5,774 patients) evaluated the impact of MRAs on the event of a stroke. The role of MRAs in stroke occurrence was not statistically significantly different (RR 1.10, 95% CI 0.82–1.47), with no heterogeneity (*I*^2 ^= 0%, *p* = 0.53) (Supplementary Figure S6). The corresponding NNTH is 341 (95% CI, NNTB 189 to ∞ to NNTH 72).

#### 3.2.6. Hyperkalemia

Seven trials have reported data on MRAs for hyperkalemia, including 9,056 patients. Hyperkalemia was more common with MRAs (RR 2.06, 95% CI 1.78–2.39) than without MRAs treatment, with no heterogeneity (*I*^2 ^= 0%) (Supplementary Figure S7), NNTH 18 (95% CI, NNTH 14 to NNTH 25).

#### 3.2.7. Gynecomastia

Seven trials including 9,048 patients published the effectiveness of treatment with MRAs on improving gynecological inflammation in this population of patients. The outcomes were like those for hyperkalemia, with an NNTH of 52 (95% CI, NNTH 13 to NNTH 725) for gynecomastia in patients randomized to MRAs and placebo treatment. There was a serious level of heterogeneity (*I*^2^ = 83%). When comparing MRA drug types in groups, spironolactone was found to produce more gynecomastia [RR 7.48 (4.42–12.68)] compared to eplerenone [RR 0.72 (0.32–1.61)] (Supplementary Figure S8).

#### 3.2.8. Meta-regression analysis and publication bias

There was no proof that the noticed impact of MRA on MACE contrasted across preliminary subgroups characterized by baseline characteristics, for example, type of MRA, mean age, year of publication, mean follow-up time, level of male members, and BMI (Supplementary Table S2). Examination of the funnel plot for every result aside from gynecomastia showed no critical unevenness (Supplementary Figure S9), as has been demonstrated by the Egger test (Supplementary Figure S10).

## 4. Discussion

This paper assesses the effectiveness and safety of MRA in patients with HF through the calculation of NNT. Seven studies compared MRAs with placebo in patients with HF and provided better help with the practical application of these medications through the calculation of NNT. MRAs reduced MACE, all-cause mortality and cardiovascular death, with NNTs of 37, 28 and 34 for MRAs preventing one case over 2.1 years of treatment, respectively. MRAs increase the risk of both hyperkalaemia and gynaecomastia, with 18 and 52 patients with HF being treated for one patient to develop these complications, respectively, over 2.1 years of treatment. These results may might assume a significant part in the medical decision-making of doctors and the development and implementation of related policies.

The action of MRAs in preventing aldosterone through competitive association with mineralocorticoid receptors is proving to be an effective complementary drug to ACEi for patients with HF, thus making MRAs one of the four classes of drugs used in the treatment of heart failure (26). MRAs also reduce NP levels and LA volume in patients with HF and have mild diuretic properties, the addition of MRAs can help with diuresis in addition to the significant cardiovascular benefits in patients with HF (27). In patients with HFrEF and NYHA class II to IV symptoms, MRA (spironolactone or eplerenone) is recommended to reduce morbidity and mortality if eGFR > 30 ml/min/1.73 m^2^ and serum potassium < 5.0 mEq/L. MRA treatment still has a high economic value compared to SGLT2i. In contrast, the benefits of MRA for HFrEF cover a wide range of etiologies and disease severity. Use in an outpatient or hospital environment is very common. Although the addition of SGLT2i to MRA therapy reduces the risk of MACE, the fact that different drugs do not have the same targets of action and therapeutic pathways does not mean that MRA therapy is ineffective (28). The role of MRA in reducing all-cause mortality compared to SGLT2i is also well studied.

This study found that the utilization of MRAs was related to a significant decrease in adverse cardiovascular outcomes, as opposed to some studies (25, 29). This may be related to the baseline risk of the selected group of people for the trial, or it may be related to the choice and dose of the drug. The binding ability of spironolactone to MR receptors is similar to that of aldosterone, but binding to the receptor inhibits its activity, resulting in an antagonistic effect. The combination of spironolactone with mineralocorticoid receptors is unstable due to the lower ability of sulfhydryl groups to form hydrogen bonds than hydroxyl groups. In contrast, non-steroidal MRAs have more selective and stable antagonistic effects due to their unique mechanism of action, and their application is more favorable with relatively fewer side effects (30). Although some studies suggest that non-steroidal MRAs (finerenone) may be as effective as steroidal MRAs in patients with HF with fewer side effects (31, 32), because the number and duration of studies of non-steroidal MRAs in cardiovascular settings are currently low, no studies of non-steroidal MRAs were included as part of search studies. Therefore, the reliability of this section needs to be improved and needs to be validated by a more definitive assessment of the results.

Studies have shown that MRAs increase the incidence of hyperkalemia, which is in agreement with previous studies (33, 34). This also limits the use of MRAs in the clinic, especially when administered externally to patients with concomitant renal dysfunction (35). Recent data have shown statistically significant clinical benefits of MRA treatment in reducing mortality in both CHF patients. This suggests that although MRA increases the incidence of hyperkalemia, it appears to protect patients from death more than patients with hyperkalemia with similar baseline characteristics and without MRA (36). And although discontinuation after the development of hyperkalemia is related to a reduced risk of repetitive hyperkalemia, the danger of mortality or cardiovascular events is higher, so the need to discontinue MRA therapy should be critically assessed (37).

In addition, we found that MRAs also produce anti-androgen-like effects, with steroidal MRAs being more likely to cause gynecological inflammation, probably because spironolactone in steroidal MRAs is less selective for mineralocorticoid receptors and also binds to sex hormones (38). These side effects make it limited in practical application. It is important to mention here that experiments on non-steroidal MRAs did not show evidence of gynecological inflammation. However, given the relatively few numbers of experiments incorporated into the analysis, the findings may not be sufficient to draw firm conclusions. More trials on non-steroidal MRAs ought to be analyzed later on to produce more statistically significant results. Therefore, when selecting drugs, we should also regulate their use from an individual perspective and consider the basic characteristics of the patient to maintain a balance between benefits and risks.

This paper is clear and unambiguous compared to other meta-analyses analyzing MRA drugs, mainly compared to placebo and in the form of NNT. The main strengths of this study are that it considered the effect of CER and that NNT expresses efficacy through a combination of baseline risk and treatment risk reduction, providing an advantage over RR. NNT is more useful than relative risk because it can tell doctors and patients more concretely, through numbers, how much effort must be put into preventing outcomes. We standardized the results for trials with different follow-up times to make the results comparable. Finally, the included trials were all placebo-controlled, reflecting the actual efficacy of the drug and ensuring that the results derived herein are not interfered with by other drugs.

There are some limitations to this paper. Firstly, the meta-analysis is an analysis of data from seven trials, not an individually based analysis of data from all those patients included in the trial. Consequently, we were unable to dependably analyze the relationship of certain variables with the noticed endpoints. Second, NNT is firmly connected with baseline risk, but the baseline risk varies among trials. According to the guidelines, previous trials have typically excluded patients with serum potassium levels >5.0 mmol/L and eGFR <30 ml/min, but due to the higher risk of patients, it is not clear that these individuals may benefit more from MRA if the hyperkalemia can be controlled.

In conclusion, this study showed that prevention of MACE in one patient with HF required treating 37 patients with HF with MRAs for 2.1 years. MRAs reduced MACE, all-cause mortality, and cardiovascular death, but no effect of MRAs on myocardial infarction and stroke was observed. MRA leads to an increment in the incidence of hyperkalaemia and gynaecomastia, with corresponding NNTs of 18 and 52.

## Data Availability

The original contributions presented in the study are included in the article/**Supplementary Material**, further inquiries can be directed to the corresponding author.
