# Supplementary material for: Mineralocorticoid receptor antagonists for chronic heart failure: a meta-analysis focusing on the number needed to treat
Source: Front Cardiovasc Med. 2023 Nov 6;10:1236008. doi: 10.3389/fcvm.2023.1236008 (PMC10657990; doi:10.3389/fcvm.2023.1236008)
Supplement: Supplementary file 1 [file Table1.docx]

Supplementary Material

# Mineralocorticoid receptor antagonists for chronic heart failure: a meta-analysis focusing on the number needed to treat

**Chang Geng, Yu-Cheng Mao, Su-fen Qi, Kai Song, Hong-Fei Wang,Zi-yan Zhang and Qing-Bao Tian***

* **Correspondence:**

Qing-Bao Tian

[tqb1980@hebmu.edu.cn](mailto:tqb1980@hebmu.edu.cn)

1. **Supplementary Table 1. The pooled CERs of MRAs.**

| Outcomes | Drug type | CER (control event rate) |  |
| --- | --- | --- | --- |
| major adverse cardiovascular events | MRA | 0.192 |  |
|  |  |  |  |
| all-cause mortality | MRA | 0.198 |  |
| cardiovascular death | MRA | 0.16 |  |
| myocardial infarction | MRA | 0.033 |  |
| stroke | MRA | 0.029 |  |
| hyperkalemia | MRA | 0.051 |  |
| gynecomastia | MRA | 0.006 |  |

1. **Supplementary Table 2. Univariate meta-regression analysis of MRAs on major vascular event**

| Model | Covariate | Classification | No. of Studies | *P* Value for |
| --- | --- | --- | --- | --- |
|  |  |  |  | Heterogeneity |
| 1 | Type of MRAs | Canrenone | 1 | 0.953 |
|  |  | Spironolactone | 4 |  |
|  |  | Eplerenone | 2 |  |
| 2 | Mean age, y |  | 7 | 0.215 |
| 3 | Published year |  | 7 | 0.223 |
| 4 | Men, % |  | 6 | 0.53 |
| 5 | Follow-up, y |  | 7 | 0.336 |
| 6 | SBP (mmHg) |  | 7 | 0.167 |
| 7 | DBP (mmHg) |  | 6 | 0.507 |
| 8 | BMI |  | 6 | 0.624 |

SBP, systolic blood pressure;DBP, diastolic blood pressure; BMI, body mass index.

1. **Supplementary Figure 1.**

**
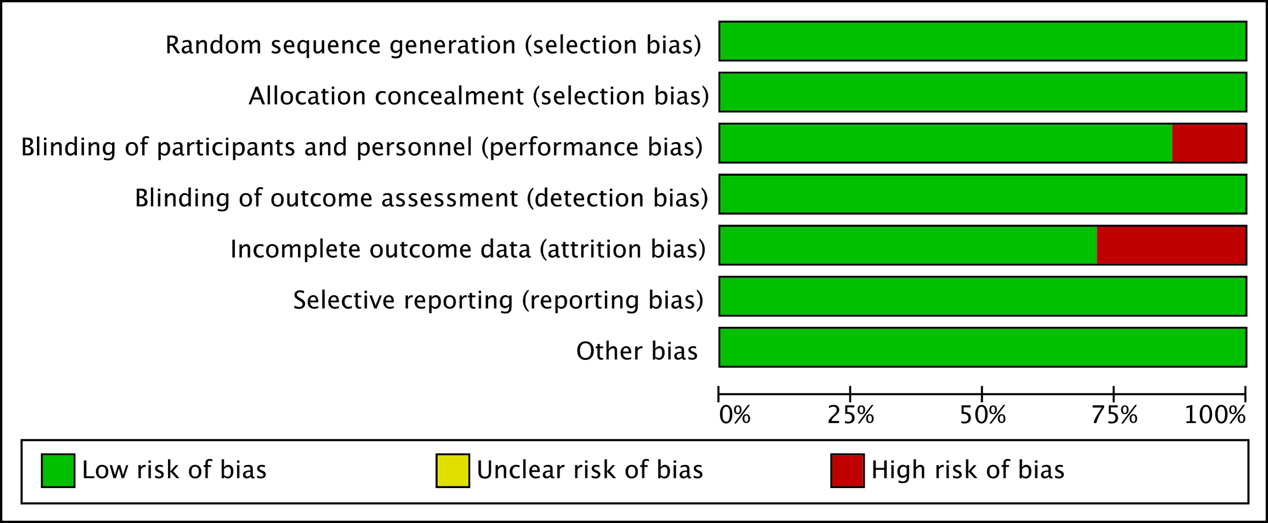
**

**
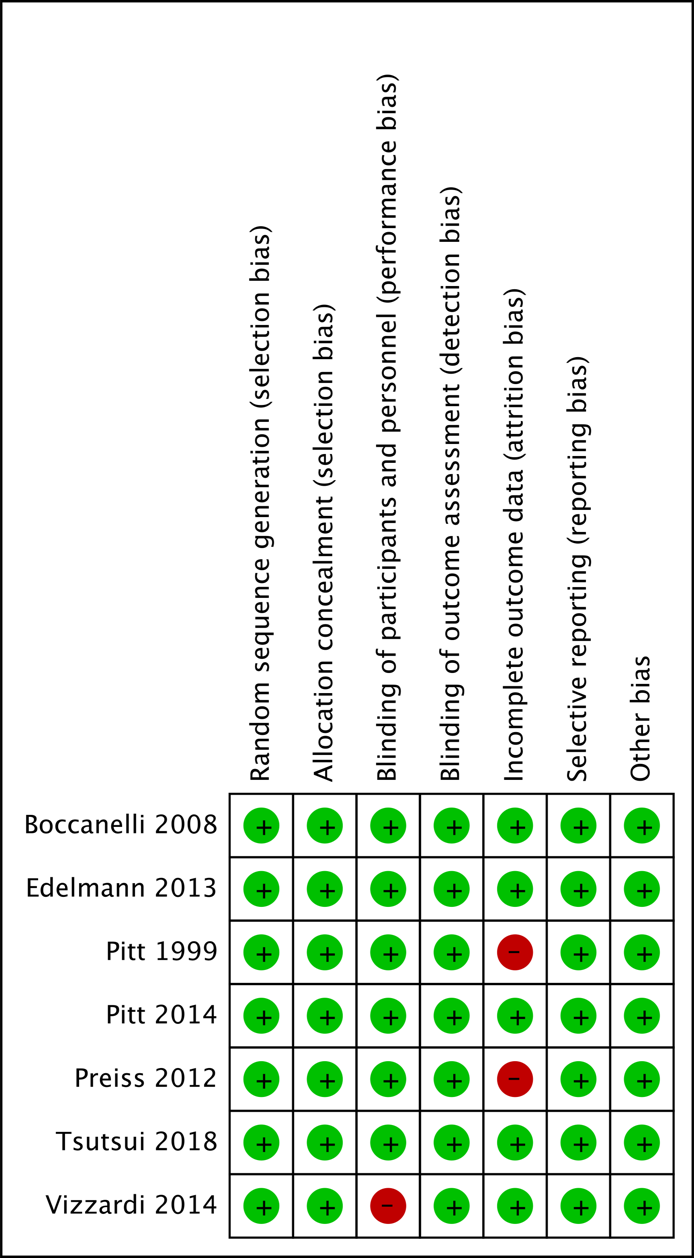
**

**Supplementary Figure 1.** Results of risk assessment.

1. **Supplementary Figure 2.**


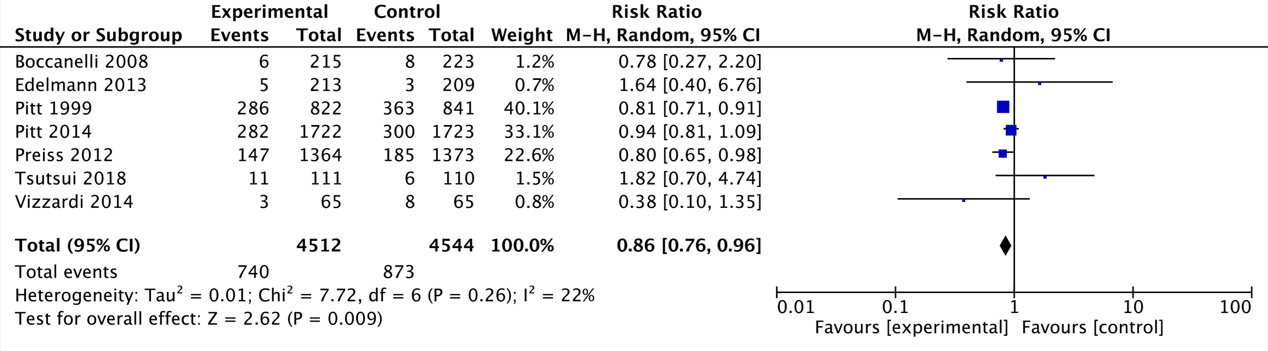


**Supplementary Figure 2.** Risk estimates using random effect models for major vascular events.

1. **Supplementary Figure 3.**


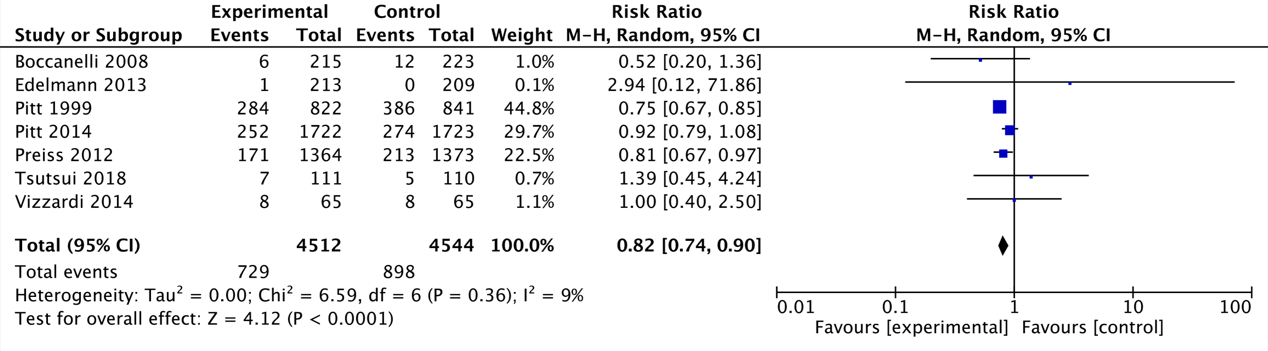


**Supplementary Figure 3.** Risk estimates using random effect models for all-cause death.

1. **Supplementary Figure 4.**


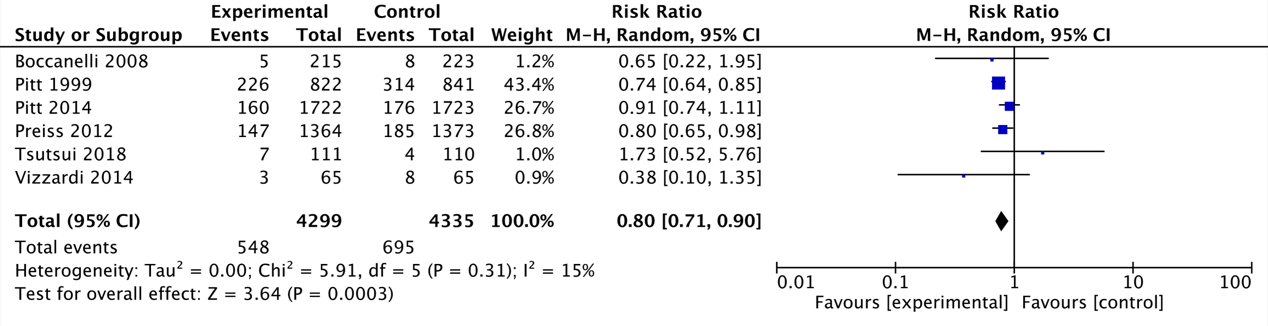


**Supplementary Figure 4.** Risk estimates using random effect models for cardiovascular mortality.

1. **Supplementary Figure 5.**


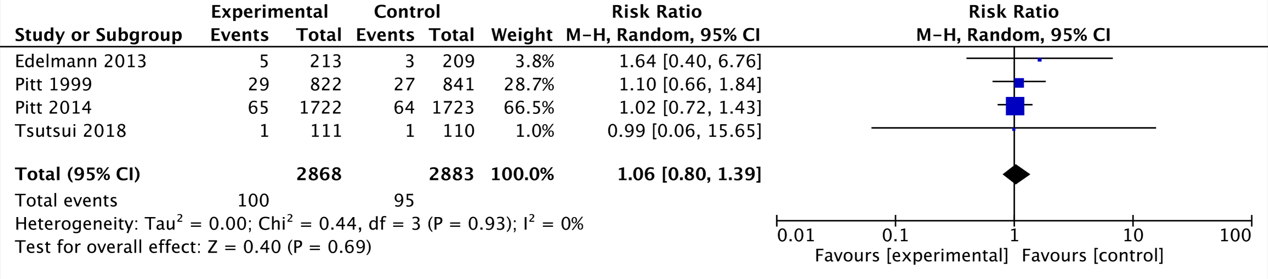


**Supplementary Figure 5.** Risk estimates using random effect models for myocardial infarction.

1. **Supplementary Figure 6.**


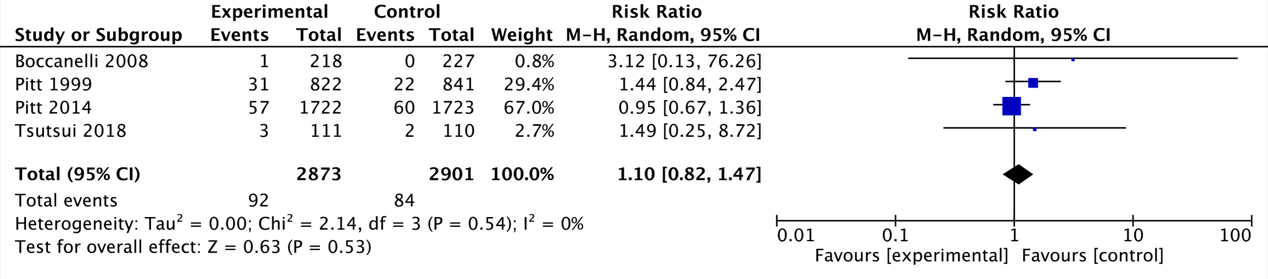


**Supplementary Figure 6.** Risk estimates using random effect models for stroke.

1. **Supplementary Figure 7.**


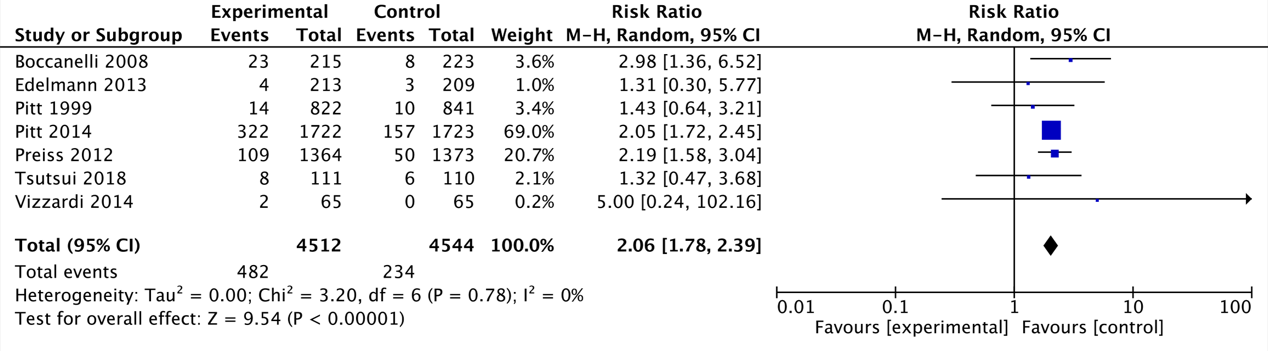


**Supplementary Figure 7.** Risk estimates using random effect models for hyperkalemia.

1. **Supplementary Figure 8.**


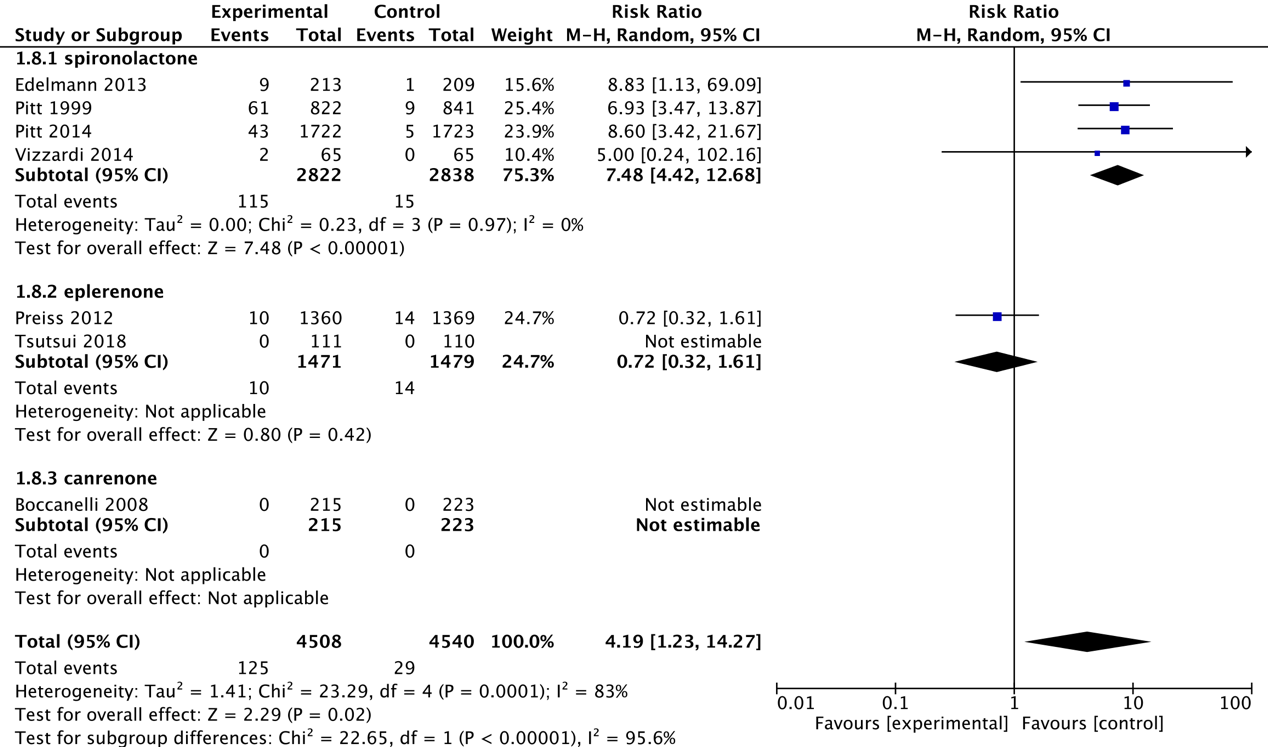


**Supplementary Figure 8.** Risk estimates using random effect models for gynecomastia.

1. **Supplementary Figure 9.**


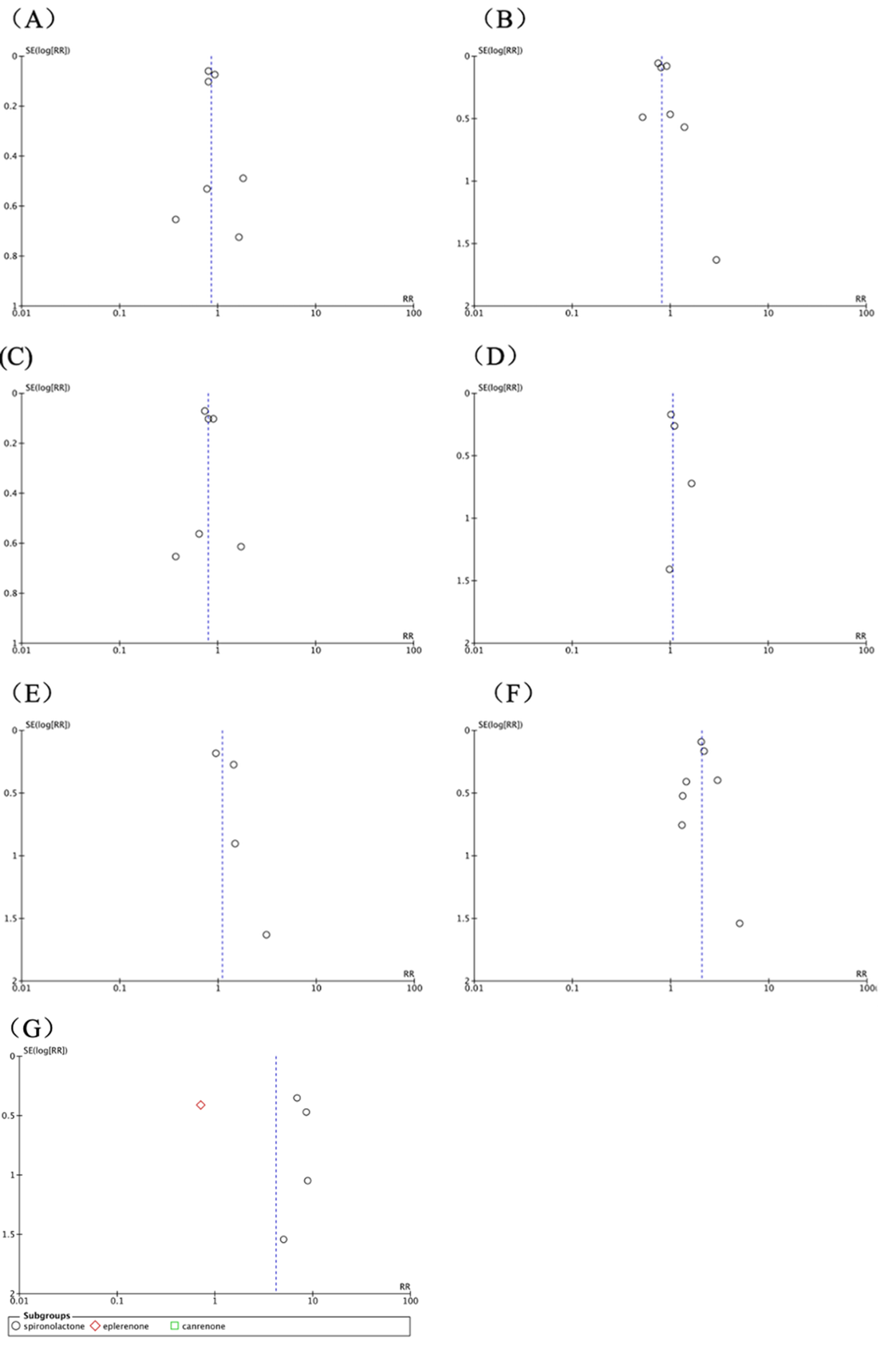


**Supplementary Figure 9.** Funnel plot analysis for major vascular events (A), all-cause death (B), cardiovascular mortality (C), myocardial infarction (D), stroke (E), hyperkalemia (F) and gynecomastia(G).

1. **Supplementary Figure 10.**


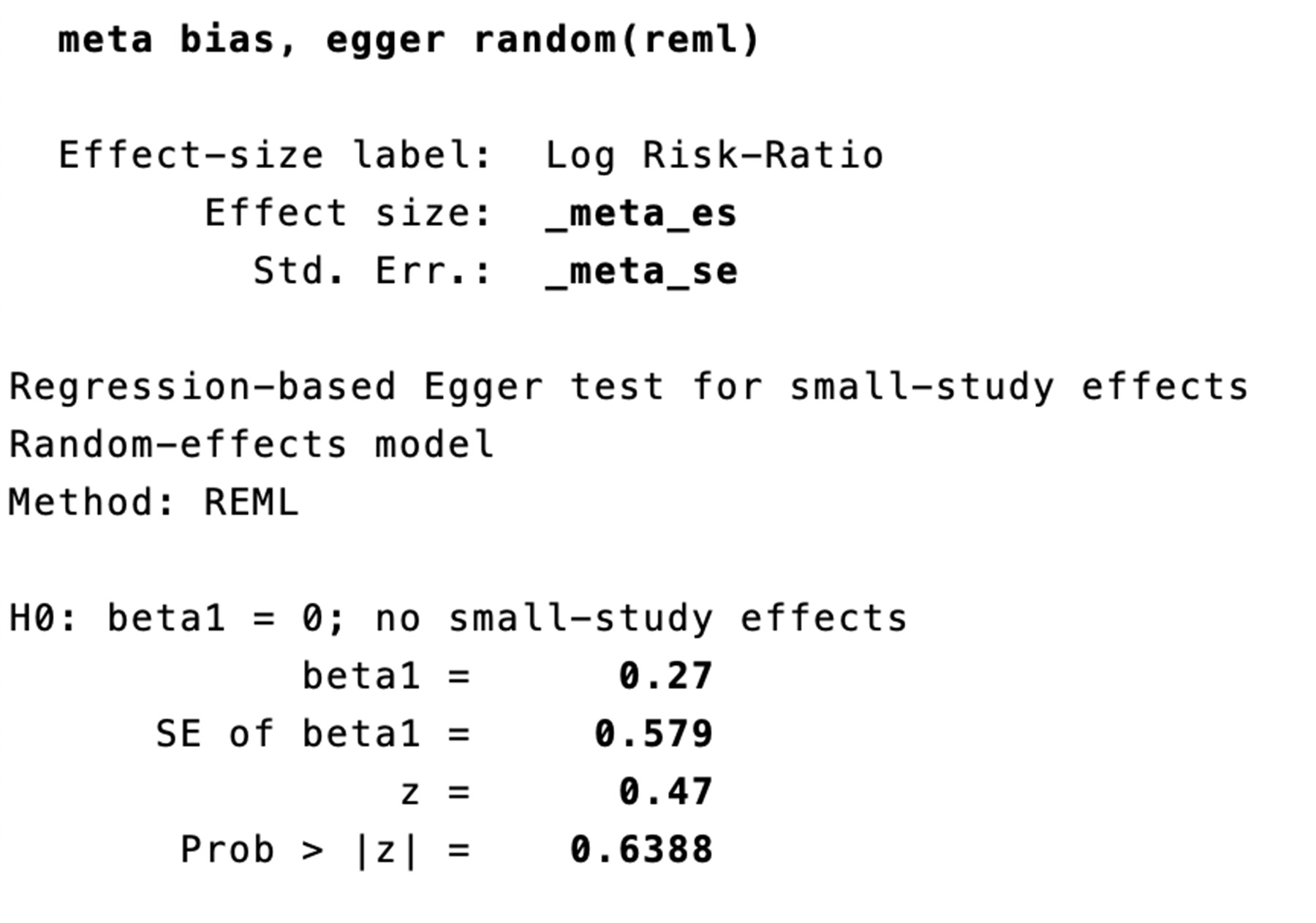


**Supplementary Figure 10.** Egger’s test.
